# Telemedicine in addictions feasibility RCT – staff and patient qualitative satisfaction

**DOI:** 10.1192/bjo.2021.786

**Published:** 2021-06-18

**Authors:** Dominic Treloar, Soraya Mayet

**Affiliations:** 1 Hull York Medical School; 2 Humber Teaching FT

## Abstract

**Aims:**

Opioid dependence has high risks. Opioid substitution treatment (OST) improves outcomes. Addiction specialist prescribers prescribe OST and monitor safety, but nonattendance may lead to worse outcomes. Telemedicine can reduce travel and improve attendance at appointments. Before COVID-19, we started a telemedicine in addictions trial to see if this helped in addictions. We present the qualitative patient and staff experience results.

**Method:**

Health Research Authority approval for randomized controlled feasibility trial of Telemedicine versus Face-to-Face (control) consultations at community addictions semirural service (2500km2) using a modified Hub-and-Spoke (outreach) model. Adult opioid dependent patients prescribed OST and attending outreach were recruited. Participants received two appointments in randomized group. Telemedicine was delivered using Skype-for-business videoconferencing. Patients attended outreach, saw keyworker for drug testing first, and telemedicine conducted via keyworker's laptop. Addiction prescribers located remotely at Hub. Post-trial research interview conducted assessing patient and staff experience of Telemedicine versus Face-to-Face consultations. Data transcribed, inputted to RedCap Cloud and free-text analysed using qualitative thematic analysis.

**Result:**

Of fifty-nine patient participants, 58 completed a research interview. Patient participants reported similar levels of satisfaction between the Telemedicine and Face to Face groups. The themes generated in relation to Face-to-Face were no difference, easy, kind staff and liking being part of research. For Telemedicine, themes were less travel, good experience, easier to access, good communication, saves time and saves money. For instance, one patient stated ‘Clear, easy to access less travel’ and another patient stated ‘I struggle with travel. I found it easier’. Of 19 staff participant research interviews completed, Staff reported Good or Very Good experience with telemedicine which was equivalent for Face-to-Face consultations. Eleven staff had experience of telemedicine consultations during the trial. They reported similar themes to patients with telemedicine leading to less travel, beneficial to patient care, improves attendance and was innovative technology. One staff member reported satisfaction with telemedicine due to ‘Time, travel and money reduction’. When questioned on the downsides to telemedicine, technological issues were mainly related to connection issues and sound issues.

**Conclusion:**

In the first known RCT of Telemedicine versus Face-to-Face consultations for patients with opioid dependence attending prescriber review, we found that both patients and staff were satisfied with telemedicine as compared to face-to-face consultations. Overall themes were reduced travel, saving time and more convenience. This will be very important given the impact of COVID-19 on access to addictions services.

Financial Sponsorship

East Riding CCG

Academic Health Science Network

